# Assessment of a Light-Curable Hydrogel to Be Used for Root Canal Obturation

**DOI:** 10.1177/00220345241287504

**Published:** 2024-12-14

**Authors:** S. Bhandari, S. Kuehne, J. Camilleri

**Affiliations:** 1 Dentistry, School of Health Sciences, College of Medicine and Health, University of Birmingham, Birmingham, UK; 2 School of Science & Technology, Nottingham Trent University, Nottingham, UK

**Keywords:** antimicrobial, sealers, material characterization, obturation degradation, microbial challenge

## Abstract

Root canal obturation involves filling of the chemomechanically prepared root canal space. Despite reduced microbial load, residual bacteria can still lead to reinfection and treatment failure. Currently, obturation techniques use a combination of gutta-percha and sealer, which requires the preparation of the root canal to specific sizes and tapers to enable the fitting of customized cones. This study aims to characterize the physical, chemical, and antimicrobial properties of a new light-curable injectable material (OdneFill, Switzerland) used to obturate the root canal. Odnefill and 2 root canal sealers (AH Plus and BioRoot RCS) were characterized by scanning electron microscopy (SEM) and energy-dispersive spectroscopy following exposure to chlorhexidine, sodium hypochlorite, and water. The flow, film thickness, radiopacity, solubility, and contact angle were evaluated. The susceptibility to microbial degradation was assessed by weight changes after contact with bacterial enzymes (lipase and cholesterol esterase). A multispecies biofilm composed of *Streptococcus mutans*, *Enterococcus faecalis*, *Fusobacterium nucleatum*, and *Veillonella dispar* was used to assess changes to the material microstructure (SEM). Further, bacterial viability in contact with the materials was evaluated using live/dead staining and confocal microscopy. A direct contact assay was carried out, and the utilization of the materials as a carbon source for the bacterial biofilm was also assessed. Statistical analysis was performed using 1-way analysis of variance and Tukey post hoc tests (*P* = 0.05). OdneFill was composed of an organic matrix with zirconium oxide filler. It exhibited comparable physical properties to AH Plus and BioRoot RCS and was stable in contact with irrigating solutions and with the bacterial enzymes (cholesterol esterase and lipase). Its antimicrobial characteristics were better than those of AH Plus when placed in contact with a multispecies biofilm. Based on the findings, OdneFill presents itself as suitable root canal–filling material and warrants further clinical investigation.

## Introduction

Solid cones composed of gutta-percha and root canal sealers are used to fill the root canal space with the aim of preventing reinfection. The root canal space has a complex morphology (Mamat and Nik Abdul Ghani 2023); therefore, preparation with standardized instruments leaves most of the root canal space unprepared (Siqueira Junior et al. 2018). Root canal irrigation and the use of sodium hypochlorite aids the elimination of bacterial biofilms and pulpal remnants, whereas calcium chelators remove the smear layer created by mechanical debridement (Zehnder 2006). Following chemomechanical preparation, the root canal space has an irregular shape and is encased with modified dentine. The micro structure of the dentine depends on the irrigation regime used (Fernandes Zancan et al. 2021), with different adjuncts resulting in deeper penetration than others (Galler et al. 2019).

The use of standardized solid cones has evolved from the standardized preparation of the root canal space with the sealer aiming at filling the remaining intricacies (Heran et al. 2019). The use of gutta-percha and sealer creates an interface between the gutta-percha and sealer and between the sealer and the root dentine. Methods of filling the root canal without the standardized gutta-percha cones will enable more conservative root canal preparation techniques whereby the root canal is not prepared to fit a standardized cone but can retain its own natural anatomy (Lussi et al. 1999; Lussi et al. 2002). The use of adequate disinfection remains of utmost importance, but the reduced use of instrumentation will also reduce the smear layer created, leading to a reduction in use of chelators as well. The aim of the current study was the assessment of the physical properties and antimicrobial characteristics of OdneFill (Odne), a photocurable filled hydrogel used to obturate root canals. The proposed method is an easy obturation technique composed of a single phase in which, after irrigation, the material will be injected into the root canal and light cured with an appropriate light source.

## Materials and Methods

The investigated materials and their composition are shown in Table 1a. The OdneFill material was dispensed through a syringe and light cured using Odne AG curing light for 120 s as instructed by the manufacturer. AH Plus and BioRoot were prepared according to the manufacturers’ instructions and allowed to set at 37 °C and 100% relative humidity for 48 and 24 h, respectively.

**Table 1a.** Chemical Composition of the Test Materials Used in the Study.

OdneFill	BioRoot RCS		AH Plus	
Paste	Powder	Liquid	Epoxide Paste	Amine Paste
Water	Tricalcium silicate	Water	Diepoxide	1-adamantane amine
Methacrylated polyethylene glycol		Polycarboxylate		N, N′-dibenzyl-5-oxa-nonandiamine-1,9
Photoinitiator (387-nm wavelength)	Povidone	Calcium chloride		TCD-diamine
Stabilizer			Calcium tungstate	Calcium tungstate
Zirconium oxide	Zirconium oxide		Zirconium oxide	Zirconium oxide
			Aerosil	Aerosil
			Pigment	Silicone oil

## Characterization and Physical Properties

### Characterization of Materials

The microstructure and elemental analysis were assessed by scanning electron microscopy (SEM; EVO MA10, Carl Zeiss Ltd.) and energy-dispersive spectroscopy (EDS; INCA, Zeiss Oxford Labs) performed on material specimens (10-mm diameter, 2-mm thickness) that were allowed to set at 37 °C and 100% relative humidity. The specimens were then embedded in resin followed by grinding and polishing.

### Characterization of the Light Source and Assessment of Degree of Conversion of Hydrogel

The methodology is given in the Appendix.

### Effect of Irrigating Solution on the Material Chemistry and Microstructure

The effect of 0.2% chlorhexidine, 5.25% sodium hypochlorite solutions compared with water on OdneFill, BioRoot, and AH Plus was assessed using a split-tooth model using human single rooted teeth described in previous research (Kapralos et al. 2022). The decoronated teeth with 1 root canal (ethical approval REC Ref 14/EM/1128), free of caries, were split and reassembled then root treated. The materials were dispensed into the root canal, and the AH Plus and BioRoot were allowed to set while the OdneFill was light cured. After 1 wk, each root was unwrapped, and the root fragments were gently detached with the use of a scalpel to expose the sealers, which were retrieved intact from the dentin walls. The material that was in contact with the irrigated dentine was characterized by SEM and EDS.

### Determination of Physical Properties of the Materials

The radiopacity, flow, film thickness, and solubility were measured according to ISO 6876;^2012^. The material hydrophilicity was assessed by the advancing contact angle method whereby a 5-µL drop of water was dispensed on the sample surface using a micro syringe, and for each specimen, 3 angle measurements were taken every 10 s. The drop volume was then slowly increased, and steps were repeated until constant contact angle measurements were achieved. The angles were measured by a specific image analysis software (OPTIMAS 6) interfaced with the camera.

## Microbiology

For the microbiology evaluations, phosphate-buffered saline (PBS; Sigma Aldrich), lipase PS enzyme (Sigma Aldrich), and cholesterol esterase enzyme (EMD Millipore Corp) were used. The enzyme powders were dissolved in 0.1 M PBS containing 0.1% sodium azide (Sigma Aldrich) to inhibit bacterial growth.

### Degradation of Endodontic Materials by Bacterial Enzymes

Weight loss analysis after contact with 3 mL of enzyme solutions, 0.15 wt/v% lipase (45 U/mL) and cholesterol esterase (40 U/mL) with pH 7 was undertaken in line with Tay et al. (2005). PBS was used as a control solution and discs not immersed in solution as a general control. The weight loss was measured to the accuracy of 0.001 g. The weight of the PBS was subtracted to make up for the liquid uptake of materials in solution. Topography was analyzed by SEM in secondary electron mode.

### Assessment of Material Changes in Contact with Bacterial Enzymes and Biofilm

Material discs were tested with bacterial species *Streptococcus mutans* (ATCC 3209), *Enterococcus faecalis* (ATCC 29212), *Fusobacterium nucleatum* (FNN-25; ATCC 25586), and *Veillonella dispar* (NCTC 11831) grown at 37 °C in an anaerobic chamber (Whitley DG250 workstation, Don Whitley Scientific Limited) for 3 d to form biofilms. As negative controls, discs (of OdneFill, AH Plus, and BioRoot) without bacteria and an empty coverslip were used. Positive controls were a coverslip with bacteria and, dependent on the experiment, discs of AH Plus and BioRoot with bacteria. For SEM analysis, a disc with an enzyme solution, cholesterol esterase in PBS, was used as an additional control. The test samples were placed in a 24-well culture plate for the biofilm assay. Overnight cultures of the bacteria were diluted to 10^3^ bacteria/mL in artificial saliva, which was then used to inoculate the discs initially (500 µL of each bacterial o/n suspension). The biofilm was allowed to form over the samples for 3 d, during which the media (artificial saliva or enzyme solutions) were changed every 24 h. After 3 d, the discs were carefully lifted into a fresh 24-well plate and stained with live/dead staining (Baclight, Invitrogen; Mountcastle et al. 2021). Biofilms were visualized and quantified using a laser scanning confocal microscope (Leica sp8 Microsystems GmbH, Mannheim, Germany). Images were taken for biofilm viability analysis using our published method (Mountcastle et al. 2021). Furthermore, a separate set of discs, incubated in the same way, were used to determine viable counts (as described below for the direct contact test). A final set of discs was prepared for and observed using SEM to analyze changes to the material after biofilm growth (*n* = 3).

### Direct Contact Test

Overnight bacterial cultures of *S. mutans* and *E. faecalis* in brain heart infusion (BHI) were diluted until 0.1 optical density using a Jen 6400 spectrometer. Twenty microliters of this suspension (containing 0.1 × 10^8^ bacteria/mL) was then pipetted onto the disc surface. An autoclaved glass coverslip (2 cm × 2 cm) was carefully positioned on top to spread the inoculum over the entire disc surface. The Petri dishes were closed, wrapped in cling film, labeled, and incubated at 37 °C in a CO_2_ 5% incubator for *S. mutans* and in a standard 37 °C incubator for *E. faecalis* for 24 h. A dry disc without bacteria, 1 disc with cholesterol esterase change, and AH Plus/BioRoot discs were used as controls. Following the incubation period, the whole disc with the coverslip assembly was immersed in 10 mL of BHI in a Falcon tube and vortexed for 1 min at full power to dislodge the surface-adhered bacteria. This was followed by the Miles and Misra method (Terleckyj et al. 1975; Alexander et al. 2021), with serial dilutions of the bacterial suspension. Of each dilution, 3 × 20 µL was pipetted into a quadrant of a BHI agar plate, and the number of colony-forming units (CFUs) at each dilution was recorded after overnight incubation at 37 °C. The number of colonies per milliliter was then calculated. The assay was run in triplicate.

### Statistical Analysis

The statistical analysis was performed using GraphPad Prism version 9.5.0. The data were tested to ensure they were a normally distributed analysis of variance with *P* = 0.05. One-way analysis of variance was then used to determine whether there were significant differences between data sets, and Tukey post hoc tests were used to determine the difference between the groups.

## Results

### Characterization of Materials

**Figure 1.**
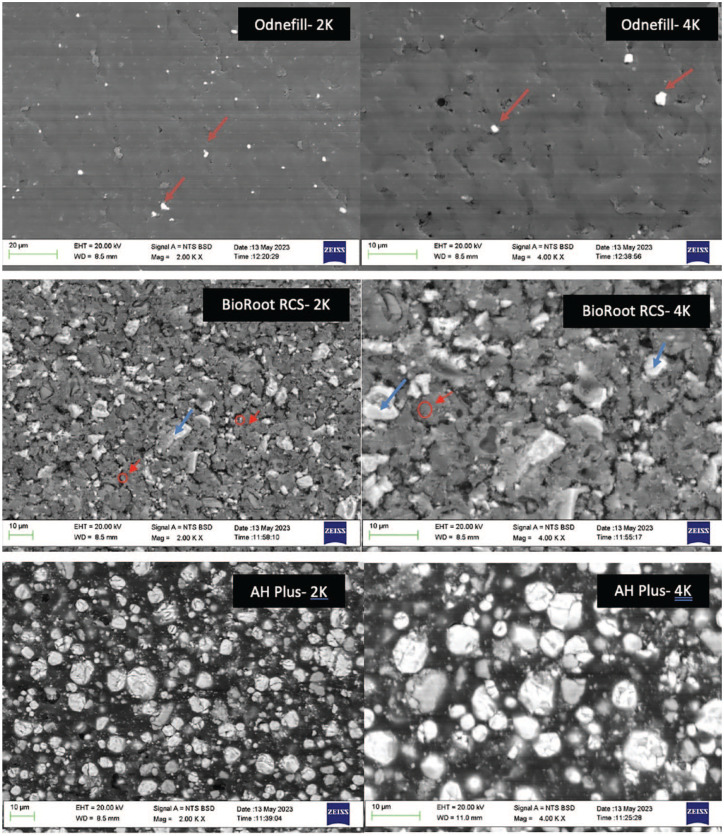
Back scatter scanning electron micrographs of all sealers showing the microstructural characteristics (magnification ×2K, ×4K). The red arrows in OdneFill SEM show the zirconium oxide particles, the red circles in BioRoot RCS show the unhydrated cement particle, and the red arrows show the hydration product forming a halo around the cement particle; The blue arrows in BioRoot RCS show the zirconium oxide particles.

The SEM images of OdneFill exhibited a smooth microstructure with deposits over the surface, which included radiopaque circular structures that were rich in zirconium (Fig. 1a, red arrows). The higher-magnification micrograph showed some porosity. The material elemental composition is shown in Table 1b. The amount of zirconium was higher in OdneFill compared with AH Plus and BioRoot RCS (Table 1b).

**Table 1b.** Semi-quantitative Analysis of All Test Materials Showing the Main Elemental Composition in Weight Percentage.

Sealer	Calcium	Silicon	Zirconium	Tungsten	Chlorine	Carbon	Oxygen
OdneFill	/	/	38.1 ± 1.6	/	1.0 ±0.1	31.5 ± 0.2	28.3 ± 0.3
BioRoot RCS	23.2 ± 3.9	6.7 ±1.2	15.9 ± 3.2	/	1.8 ± 0.3	23.6 ± 1.5	37.2 ± 4.8
AH Plus	3.5 ± 0.2	/	13.4 ± 0.7	17.7 ±1.3	/	50.2 ± 1.0	15.3 ±0.2

BioRoot RCS exhibited a microstructure (Fig. 1a) composed of dark gray particles, some of which had a halo of lighter gray around them (cement particle in red circle and halo with red arrow). These particles were composed of calcium, silicon, and oxygen. Lighter and shinier particles (blue arrows) rich in zirconium were also present. The material also exhibited porosity and some cracks. The elemental analysis showed the presence of calcium, silicon, zirconium, and oxygen (Table 1b).

AH Plus was composed of a rounder particle morphology of different sizes and opacity. The larger particles were rich in calcium and tungsten, while the smaller ones were composed of zirconium and oxygen (Fig. 1a).

### Characterization of the Light Source and Assessment of Degree of Conversion of Hydrogel

The results for the work undertaken are given in the Appendix and shown in Appendix Figures 1 and 2.

### Effect of Irrigating Solution on the Material Chemistry and Microstructure

**Figure 2.**
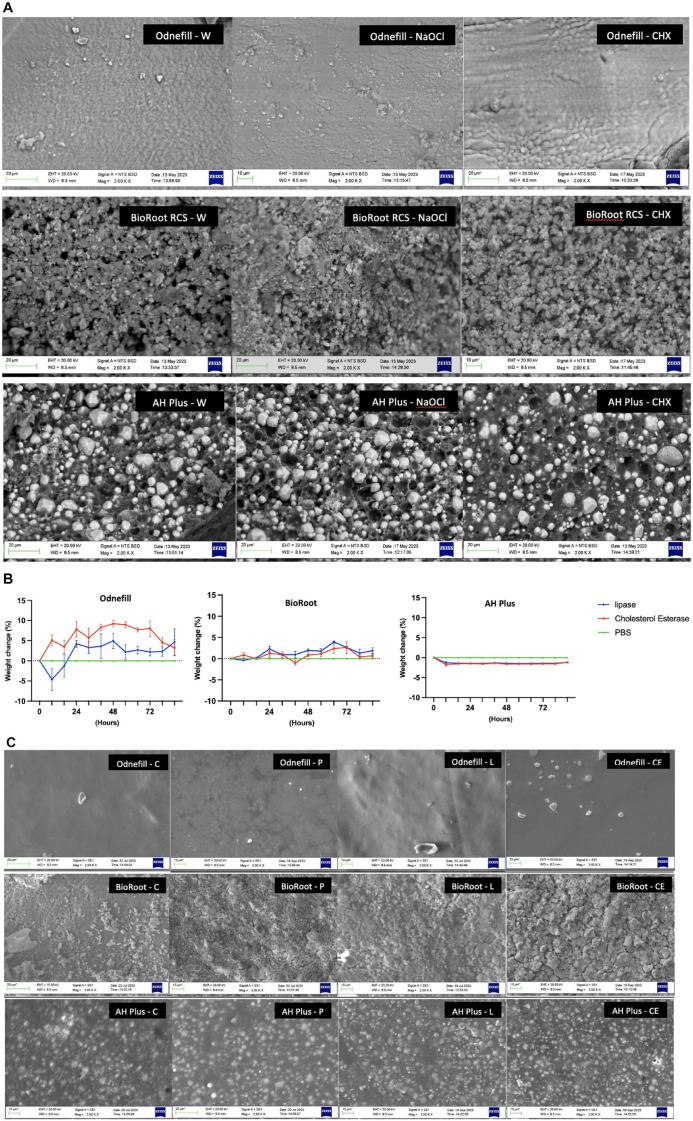
(**A**) Scanning electronic microscopic images of OdneFill, BioRoot RCS, and AH Plus irrigated with water (W), sodium hypochlorite (NaOCl), and chlorhexidine (CHX). (**B**) Percentage weight change analysis after enzymatic degradation of OdneFill, BioRoot RCS, and AH Plus after treatment with lipase enzyme, cholesterol esterase enzyme, phosphate-buffered saline (PBS), and material disk without any treatment (control) incubated at 37 °C for 3 d, changing the solution every 4 h/d. The positive *y*-axis shows a weight gain, while the negative *y*-axis shows a weight loss (*P* < 0.001). The values of lipase and cholesterol esterase are subtracted from the value of PBS. (**C**) Secondary scanning electron microscopy (SEM) images at 2k magnification of OdneFill, BioRoot RCS, and AH Plus treated for 72 h with lipase (L), cholesterol esterase (CE), PBS (P), and the control untreated materials (C) showing microstructural changes as a result of immersion in solutions.

The material microstructure and elemental analysis of OdneFill and AH Plus did not change in contact with different irrigating solutions (Fig. 2a). When compared with the sealer not exposed to solution, in Figure 1a, the AH Plus exhibited filler plucking when in contact with all 3 solutions. The BioRoot RCS exhibited microstructural changes in contact with sodium hypochlorite and chlorhexidine. There was a reduction in the zirconium content (Table 1c) in contact with sodium hypochlorite and a reduction in both calcium and zirconium in contact with chlorhexidine.

**Table 1c.** Semi-quantitative Analysis of Elemental Distribution of Materials in Percentage Atomic Weight after Contact with Water, Sodium Hypochlorite, or Chlorhexidine.

Sealer	Irrigating Solution	Calcium	Silicon	Zirconium	Tungsten	Chlorine	Carbon	Oxygen
OdneFill	Water	0.5 ± 0.1	/	43.2 ± 2.0	/	0.7 ± 0.1	28.7 ±1.0	26.7 ± 1.5
	NaOCl	1.3 ± 0.2	/	42.9 ± 5.4	/	0.7 ± 0.0	22.2 ± 1.4	32.2 ± 3.7
	CHX	1.0 ± 0.4	/	48.9 ± 2.2	/	1.0 ± 0.1	25.2 ±3.1	23.8 ± 1.4
BioRoot RCS	Water	20.4 ± 0.1	5.8 ± 0.1	21.0 ± 1.1	/	1.5 ± 0.1	19.9 ± 2.4	31.4 ± 1.3
	NaOCl	23.2 ± 3.9	6.7 ± 1.2	15.9 ± 3.2	/	1.8 ± 0.3	23.5 ± 1.5	37.2 ± 4.8
	CHX	15.8 ± 3.0	6.2 ± 1.5	16.3 ± 1.4	/	1.7 ± 0.6	23.9 ± 10.3	36.0 ± 5.5
AH Plus	Water	3.7 ± 0.1	/	12.7 ± 2.0	17.6 ± 1.7	/	46.7 ± 1.3	19.0 ± 1.3
	NaOCl	3.5 ± 0.2	/	13.4 ± 0.7	17.7 ± 1.3	/	50.2 ± 1.0	15.3 ± 0.2
	CHX	3.4 ± 0.1	/	8.5 ± 1.1	18.7 ± 0.9	/	49.3 ± 0.3	20.0 ± 0.5

### Determination of Physical Properties

The BioRoot RCS and AH Plus exceeded the minimum value specified in the ISO norm for flow, while the OdneFill flow could not be measured as it was wider than the size of the plates used (Table 1d). A second trial was undertaken with 80-mm × 80-mm plates, which is double the minimum recommended by the ISO 6876;^2012^, but the material was still out of range. Both OdneFill and AH Plus had a film thickness <50 µm, which complied with the ISO norm, while BioRoot RCS had a film thickness of 51.3 µm, which was slightly higher than the 50 µm specified in the standard. All materials had a radiopacity greater than 3 mm aluminum thickness.

**Table 1d.** Results of Physical Properties of the Test Materials and the ISO Norm Indicating Whether the Materials Are within Range.

Material	Property				
	Flow, mm	Film Thickness, µm	Radiopacity, mm Al	Solubility, %	Contact Angle, °
Odnefill	Out of range	3.0 ± 0.0	3.3 ± 0.3	5.5 ± 0.1	41.5 ± 2.7
BioRoot RCS	21.1 ± 5.3	51.3 ± 6.6	3.0 ± 0.2	15.9 ± 0.9	21.1 ± 2.7
AH Plus	21.7 ± 2.0	18 ± 6.5	10.6 ± 0.4	0.1 ± 0.07	71.2 ± 3.5
ISO norm	>17	<50	>3	<3	/

All materials had a contact angle less than 90° with AH Plus being the least wettable surface. Low-contact angles signify hydrophilicity. The BioRoot RCS was the most hydrophilic and AH Plus the most hydrophobic material.

### Degradation of Endodontic Materials by Bacterial Enzymes

There was an initial loss in weight for OdneFill when incubated with lipase and a sustained weight loss for AH Plus in both enzyme solutions (Fig. 2b). OdneFill and AH Plus did not show any microstructural changes in contact with the enzyme solutions (Fig. 2c). OdneFill exhibited some surface changes in contact with PBS. The BioRoot control had an aggregation of circular crystals on its surface, which is in keeping with the surface carbonation reported previously over the surfaces of hydraulic cements. This is caused by a reaction of the calcium hydroxide produced as a by-product of hydration to the atmospheric carbon dioxide (Camilleri et al. 2004; Camilleri et al. 2005). Immersion in solution produced varying levels of surface carbonation with the least shown in PBS and the most extensive in the cholesterol esterase.

### Assessment of Material Changes in Contact with Bacterial Enzymes and Biofilms

**Figure 3.**
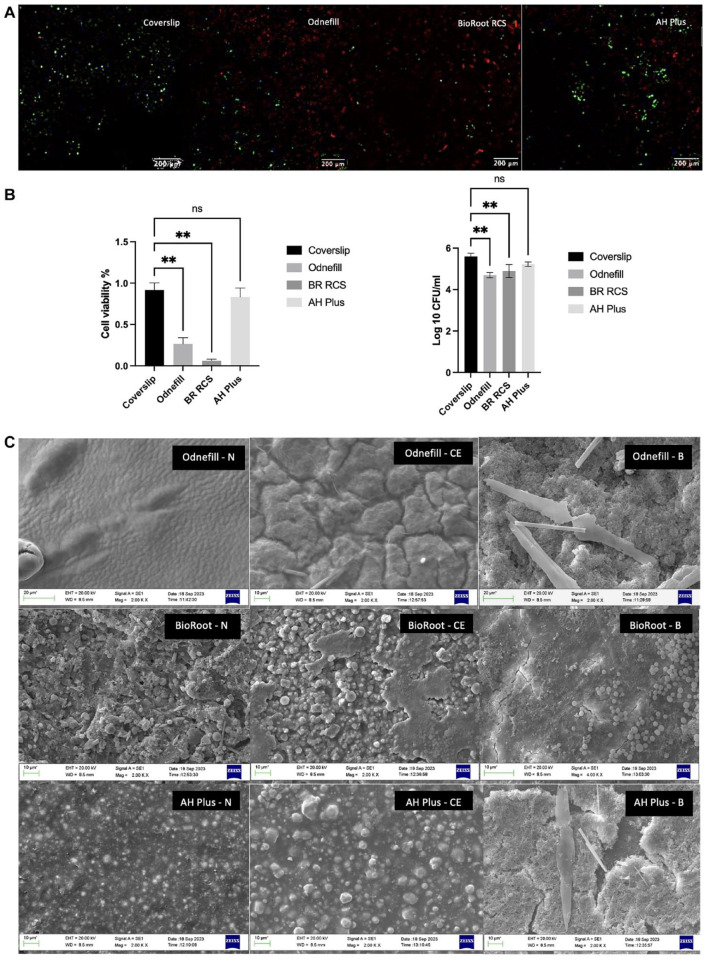
(**A**) Confocal scanning images of 3-d multispecies biofilms in contact with the different materials compared with the coverslip. Green signals indicate live cells, and red signals indicate dead cells (scale bar: 200 µm). (**B**) Comparison of cell viability percentages and log 10 of the CFU/mL in 3-d biofilms of *Fusobacterium nucleatum* ssp *nucleatum* (FNN-25), *Veillonella dispar*, *Streptococcus mutans*, and *Enterococcus faecalis* on AH Plus, BioRoot RCS, OdneFill, and the coverslip with bacteria, with mean ± standard deviations. Five confocal images were analyzed of each of 3 biological replicates (*n* = 3). ImageJ macro was used to obtain the percentage viability of cells (*P* ≤ 0.01). (**C**) Scanning electron microscopy (SEM) images in secondary electron mode at 2k magnification of the test materials, OdneFill, BioRoot RCS, and AH Plus, after contact with cholesterol esterase (CE) and a 3-d multispecies biofilm (B) of *F. nucleatum* ssp *nucleatum* (FNN-25), *V. dispar*, *S. mutans*, and *E. faecalis*, compared with the materials (N) not exposed to enzymes or bacteria. Note: BioRoot-B is in higher magnification as compared with the others. **P> 0.01.

The control biofilm showed mostly green fluorescence (Fig. 3a), indicating that the bacteria were viable throughout the experimental period. Both OdneFill and BioRoot RCS exhibited reduced bacterial growth/biofilm formation, with BioRoot RCS showing higher reduction in bacterial load (Fig. 3b). AH Plus was similar to the control (*P* > 0.05). The results of CFUs showed a similar trend (Fig. 3b). The scanning electron micrographs show changes to OdneFill and AH Plus surfaces in contact with cholesterol esterase and the biofilm with the changes more marked in the latter (Fig. 3c). The cholesterol esterase led to the formation of ridges, resulting in a rough surface of the OdneFill. This was also observed in AH Plus, where the radiopacifier particles were more evident after contact with cholesterol esterase, indicating some degradation. Biofilm was observed on both OdneFill and AH Plus covering the whole material surface. BioRoot RCS exhibited carrying degrees of carbonation on its surface for all specimens.

### Direct Contact Test

**Figure 4.**
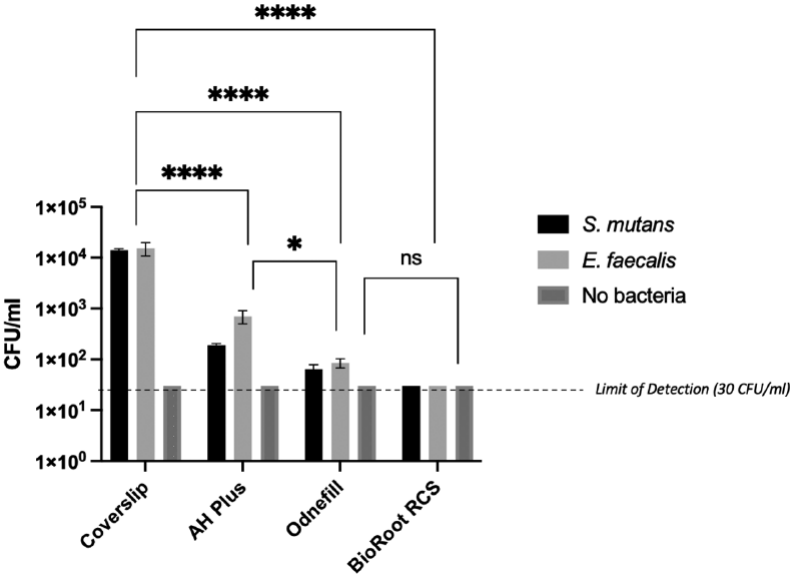
Antimicrobial activity in CFU/mL of 3 different sealers (AH Plus, OdneFill, and BioRoot RCS) and a coverslip after 24 h of direct contact test with *S. mutans* and *E. faecalis*. Results of 2-way analysis of variance test with *P* < 0.0001: statistically significant difference. The lowest limit of detection was at 30 CFU/mL. ****P > 0.0001 and *P > 0.1.

Figure 4 shows the results of the direct contact test. BioRoot RCS did not exhibit any bacterial growth with all groups (*P* < 0.001 compared with control). Both OdneFill and AH Plus reduced the microbial load for both species but to a lesser extent than BioRoot RCS did. AH Plus was least antimicrobial among all materials tested.

## Discussion

The current study investigates a novel light-curing injectable hydrogel proposed to be used as an obturating material to replace gutta-percha and sealer combinations. This technique allows for a more conservative root canal preparation. A noninstrumentation technique has been proposed in the past (Lussi et al. 1993; Lussi et al. 1999; Lussi et al. 2002), exhibiting comparable obturations clinically (Lussi et al. 2002) to root canals filled with gutta-percha different sealers (Lussi et al. 1999). Noninstrumentation may be a solution to avoid overcutting and overpreparation of the root canal, which together with irrigating solutions (Galler et al. 2019) destroys the root dentine microstructure (Karatas et al. 2023). However, obturation of the root canal will be challenging.

OdneFill is an injectable hydrogel that will enable the obturation of all root canal anatomy regardless of the preparation method. The light source was optimized to have an irradiance matching the photoinitiator in OdneFill, thus enabling an adequate cure. The polymerization at different thicknesses was assessed to optimize the clinical method. Measurement of the double bond conversion of the monomer provided inconclusive results due to the presence of water in the hydrogel, impeding precise measurement of double bond absorptions. Nevertheless, the current results demonstrate the highest degree of conversion at 2- to 5-mm sample thickness. Further studies are needed to determine precisely the degree of conversion. Notably, there is currently no comparable obturation system in clinical use; thus, 2 root canal sealers have been used as comparators.

OdneFill was characterized using various methods including SEM and EDS for microstructure and elemental evaluation. These techniques enable the visualization of the material at high magnifications and the assessment of interactions with dentine modified by irrigating solutions. Electron microscopy is also a useful tool to assess the presence of bacteria over a material surface. The main limitation in the latter is the high magnification, thus limiting the field of view and also the inability to detect whether the bacteria are viable.

OdneFill exhibited physical characteristics comparable to both BioRoot RCS and AH Plus. Any nonconformity is acceptable as the material is not a sealer. Furthermore, it is also command cured; thus, extrusion past the apex is not high risk, as it can be cured immediately. The contact angle was in the middle range between AH Plus and BioRoot RCS, thus allowing the material to retain itself within the confines of the root canal. The radiopacity was within range, indicating that OdneFill is a suitable root canal–filling material possessing all the prerequisite properties. The solubility was higher than the ISO specifications. The solubility testing of root canal sealers was undertaken using a gravimetric method with sealer discs placed in 50 mL of water. It has been shown in various studies (Kebudi Benezra et al. 2017; Camilleri et al. 2022; Ali et al. 2024) that changes to the testing methodology result in variation in the material solubility.

The final irrigating solution affects the antimicrobial properties of the obturating material (Arias-Moliz and Camilleri 2016). For hydraulic cement sealers, matching the irrigation to the obturating technique and sealer characteristics is crucial (Fernandes Zancan et al. 2021) with chelation necessary (Zancan et al. 2021). OdneFill was not affected by water, sodium hypochlorite, or chlorhexidine, which are used regularly in root canal therapy. The current method used to assess the interaction of irrigated dentine with the test materials/sealers (Kapralos et al. 2021; Kapralos et al. 2022) also included dentine in the test. Placing materials in irrigating solutions for a length of time is not clinically relevant.

The replacement of gutta-percha with a novel system has already been attempted with Resilon, which promised a monobloc technique with polycaprolactone-based points and an associated sealer (Teixeira et al. 2004). The main problem with Resilon was the degradation (Tay, Pashley, Williams et al. 2005; Tay, Pashley, Yiu, et al. 2005; Hiraishi et al. 2007), thus resulting in microbial recolonization and treatment failure (Barborka et al. 2017; Strange et al. 2019). The microbial degradation was assessed here for OdneFill because it is a replacement of the gutta-percha obturation. The OdneFill did not exhibit any degradation in contact bacterial enzymes. This difference in results could be attributed to the possibility that any deterioration of the material could have been masked by the increase in weight of the material through water uptake.

Microbes that are not affected by chemomechanical preparation may be deterred by the antimicrobial activity of root canal–filling materials, contributing to a higher treatment success rate (Bodrumlu and Alaçam 2007). BioRoot RCS has shown strong antimicrobial potential due to release of calcium ions elevating the pH of the surrounding environment as compared with set AH Plus, which has been proven to be nonantibacterial (Kapralos et al. 2018; Alsubait et al. 2019). The viability of a multispecies, endodontic biofilm was greatly reduced when grown on OdneFill and BioRoot RCS. This was confirmed by a reduced colony count. The antimicrobial characteristics of the OdneFill could potentially be associated with the presence of methacrylates in the composition (Childs et al. 2024). The degree of conversion was low; thus, the methacrylate diffuses in solution. This phenomenon needs further investigation. AH Plus did not exhibit antimicrobial activity indicated in the direct contact test. SEM was performed to double-check the findings from the direct contact test, which supported the results obtained in the previous test. A range of microbial species was used throughout the experimentation, as this allowed for better clinical translation since the endodontic biofilm is multispecies in nature.

## Conclusions

OdneFill, a new light-curable injectable hydrogel used for root canal obturation, exhibited comparable physical and antimicrobial properties to currently used root canal sealers. It was stable in contact with irrigating solutions and bacterial enzymes.

## Author Contributions

S. Bhandari, contributed to data analysis, drafted the manuscript; S. Kuehne, contributed to conception and design, critically revised the manuscript; J. Camilleri, contributed to conception and design, drafted and critically revised the manuscript. All authors gave their final approval and agree to be accountable for all aspects of the work

## Supplemental Material

sj-docx-1-jdr-10.1177_00220345241287504 – Supplemental material for Assessment of a Light-Curable Hydrogel to Be Used for Root Canal Obturation Supplemental material, sj-docx-1-jdr-10.1177_00220345241287504 for Assessment of a Light-Curable Hydrogel to Be Used for Root Canal Obturation by S. Bhandari, S. Kuehne and J. Camilleri in Journal of Dental Research
