# Supplementary material for: Assessment of a Light-Curable Hydrogel to Be Used for Root Canal Obturation
Source: J Dent Res. 2024 Dec 14;104(3):260–9. doi: 10.1177/00220345241287504 (PMC11843793; doi:10.1177/00220345241287504)
Supplement: sj-docx-1-jdr-10.1177_00220345241287504 – Supplemental material for Assessment of a Light-Curable Hydrogel to Be Used for Root Canal Obturation [file sj-docx-1-jdr-10.1177_00220345241287504.docx]

**Appendix**

**Methodology**

***Characterization of the light source***

A fibre-based UV-Vis spectrometer was used to assess the absolute spectral irradiance of the Odnefill light source (with and without fiber tip) using a UV-Vis spectrometer (USB4000, Ocean Optics, UK; n=6). The spectrometer was coupled to a 200 µm optical fibre and an opaline glass CC3 cosine corrector (3.9 mm diameter of collection area; 6 mm outer diameter; Ocean Optics, UK) which was calibrated to a National Institute of Standards and Technology (NIST) traceable light source (Mikropack DH2000/ Ocean Optics, UK). The device was positioned concentrically and directly above the opaline glass cosine corrector at distance of 0 mm and 10 mm and spectral irradiance was measured. The absolute irradiance was subsequently determined by integrating between 395 nm and 405 nm.

***Assessment of degree of conversion of hydrogel***

Specimens were placed into pre-fabricated Teflon moulds (4 mm internal diameter, 20 mm thick) and covered with a transparent cover slip placed carefully ensuring no air bubbles. ATR-FTIR spectroscopy was used to measure the degree of polymerization. For this, the decay of the peak integral of the aliphatic C=C group (C=C; 1637cm^-1^) of the bismethacrylate hydrogel compared to a reference peak at 1549 cm^-1^ was studied. were identified for as markers for polymerisation. IR absorption was measured using Fourier Transform mid-infrared spectroscopy (FT-IR ATR-mid IR, Bruker, Invenio, Spectral resolution: 8cm^-1^, Scans 32) in rapid-scan mode utilising backward and forward interferogram capture. Interferograms were captured in real-time during irradiation using the Odne light source (10s and 120s cure) at 0 mm distance from the upper surface of each specimen (n=3) and subsequently processed to obtain spectral absorbance (400-3000 cm^-1^). Data included 10s capture without light activation, 10s capture with light activation and an addition 50s after light activation.  This procedure was repeated at 2, 5, 10, 15 and 20 mm material thickness to simulate light curing inside the root canal. Degree of conversion was calculated from the processed absorption spectra using the following Equation:


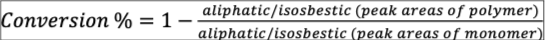
 *x 100*

**Results**

***Characterization of the light source***

The light source emitted light at a wavelength of 400 nm and the tip enhanced the irradiance as indicated in Figure 1.

***Assessment of degree of conversion of hydrogel***

Regardless of the irradiation time used (10s or 120s), the hydrogel achieved the maximum degree of conversion at 30s following the initial irradiation (Figure 2). The 2 and 5 mm thicknesses exhibited the highest degree of conversion.


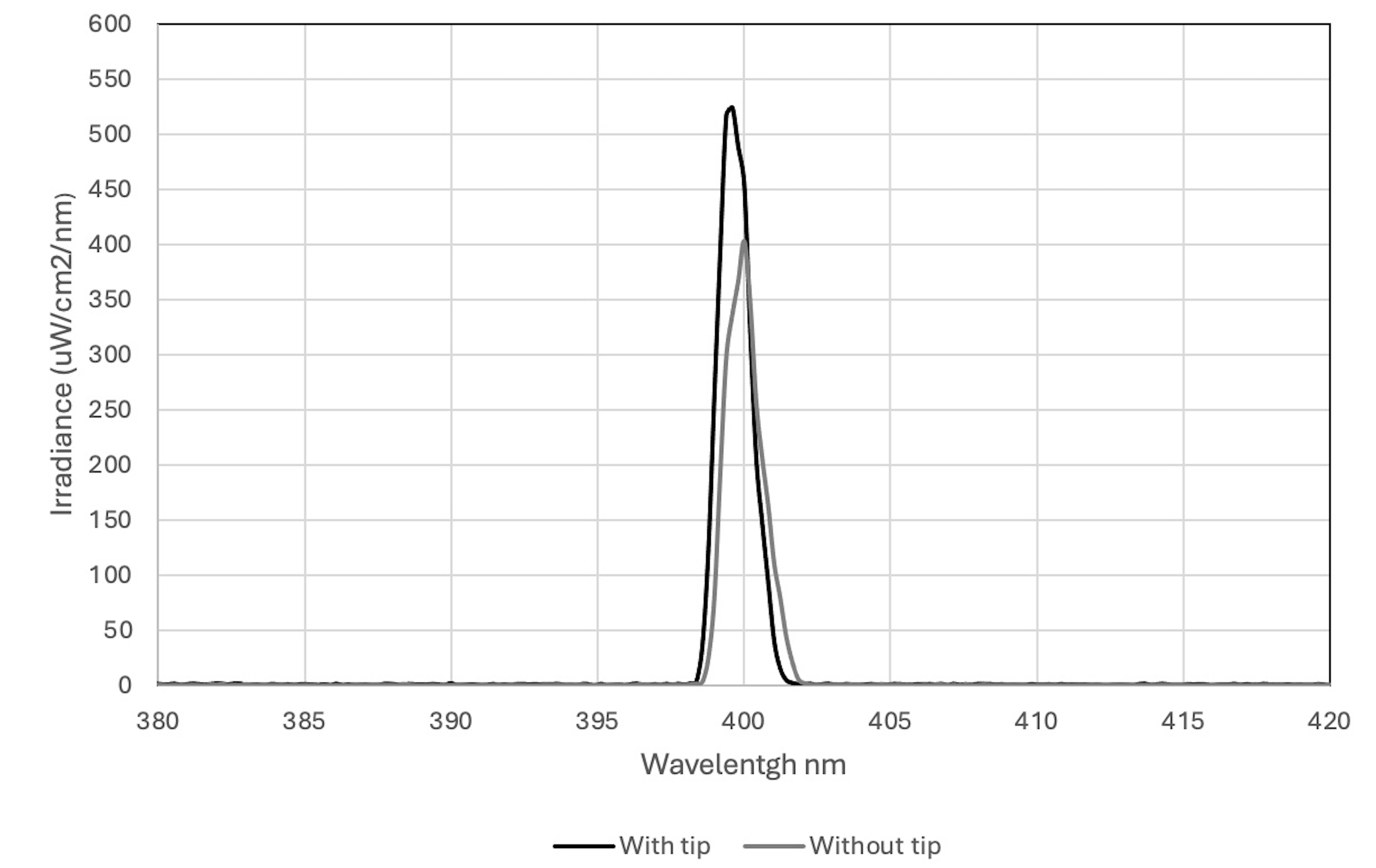
Figure 1: Irradiance achieved with the Odne light curing unit showing a peak at 400 nm with higher irradiance when using the filter.


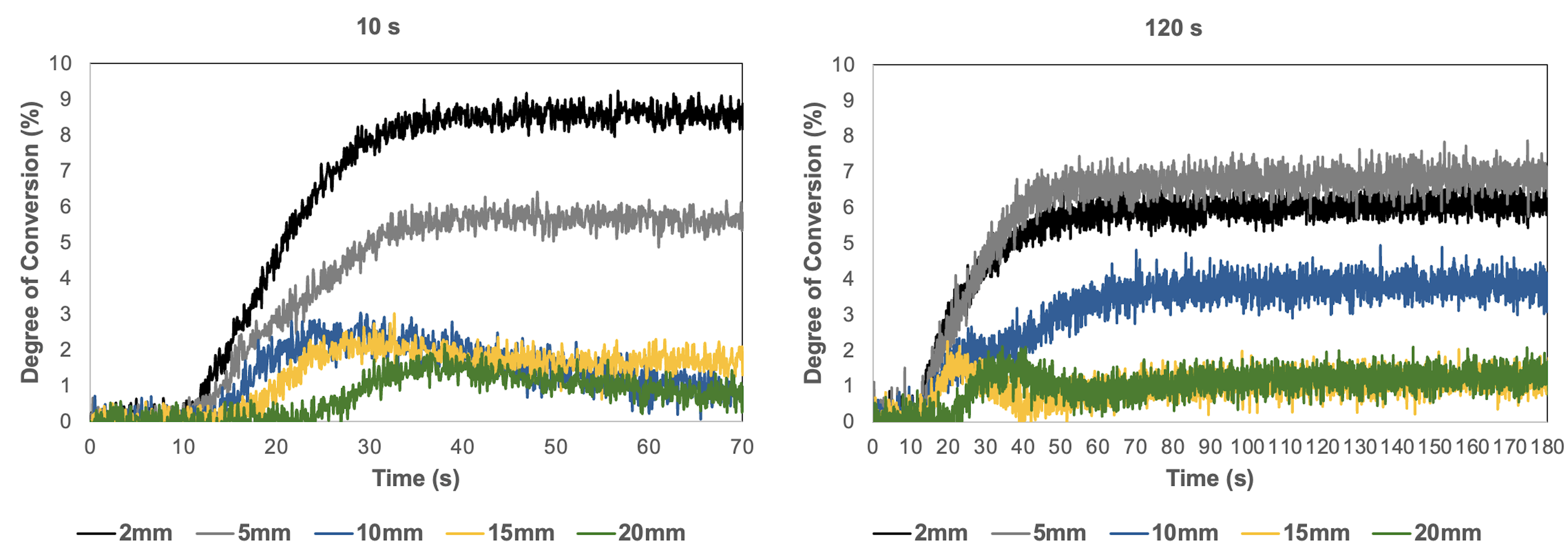


Figure 2: Degree of cure of the Odnefill at different thicknesses and 10s and 120s light exposure showing higher cure at low thickness of material
